# The Effect of Concentration on the Cross-Linking and Gelling of Sodium Carbonate-Soluble Apple Pectins

**DOI:** 10.3390/molecules24081635

**Published:** 2019-04-25

**Authors:** Diana Gawkowska, Jolanta Cieśla, Artur Zdunek, Justyna Cybulska

**Affiliations:** Institute of Agrophysics, Polish Academy of Sciences, Doświadczalna 4, 20-290 Lublin, Poland; d.ganczarenko@ipan.lublin.pl (D.G.); j.ciesla@ipan.lublin.pl (J.C.); a.zdunek@ipan.lublin.pl (A.Z.)

**Keywords:** pectin, cross-linking, pectin concentration, dynamic light scattering, AFM nanostructure, viscosity, hydrogen bond

## Abstract

The cross-linking and gelation of low-methoxy pectins are basic processes commonly used in different industries. The aim of this research was to evaluate the cross-linking process of the sodium carbonate-soluble pectins (named DASP) extracted from apples, characterized by a low degree of methylesterification as a function of its concentration in water (C_DASP_). The cross-linking process was studied with a dynamic light scattering method, atomic force microscope (AFM), viscosity and pH measurements. An increase in C_DASP_ above 0.01% resulted in a decrease in the aggregation index (AI) and the change of its sign from positive to negative. The value of AI = 0 occurred at C_DASP_ = 0.33 ± 0.04% and indicated the formation of a pectin network. An increase in C_DASP_ caused the changes in viscosity of pectin solutions and the nanostructure of pectins spin-coated on mica observed with AFM, which confirmed results obtained. The hydrogen bonds were involved in the cross-linking process.

## 1. Introduction

Pectins are polysaccharides characterized by a complex structure and gelling properties. Their main structural unit is the α-1,4-d-galacturonic acid (GalA) unit, which is a part of three main pectin domains: homogalacturonan (HG), rhamnogalacturonan I (RGI) and rhamnogalacturonan II (RGII) [1]. Homogalacturonan contains only GalA units, in which a portion of the carboxyl groups may be methylesterified, and the degree of methylesterification (DM) expresses the percentage of these groups [2]. The acetyl groups may be also present in this pectin domain at O-2 or/and O-3 of GalA [3,4]. However, they occur mainly in the hairy region of apple pectin [5,6]. Rhamnogalacturonan I and II include GalA and monosaccharides, such as arabinose, rhamnose, galactose, and, in the case of RGII, apiose, fucose, 2-*O*-methyl-xylose, 2-*O*-methyl-fucose, and others are included [7].

The cross-linking and gelling properties of pectin are commonly used in the food industry in the production of jams and fruit jellies [8]. Pectin may also be used as a carrier of bioactive substances in drug delivery systems [9] and as a biopolymeric matrix for slow release fertilizers [10]. Due to these reasons, the investigation of pectin cross-linking and gel formation is extremely important. The determination of pectin concentration, in which its cross-linking and gelling occur, has a crucial significance in the design of many technological processes. Cross-linking and gelling are influenced by pectin structure and concentration, the presence of cross-linking agents and sugar, pH conditions, and temperature. The influence of low-methoxy pectin concentration on the gelation process was studied in the presence of calcium ions and sucrose at pH = 3.5 [11], in the presence of calcium ions at pH 5 [12], and without any salt addition at pH 3 [13,14]. Rheological measurements are commonly applied to investigate pectin gelation [15]. Cross-linking and gelling of pectin was successfully analyzed on molecular level by means of atomic force microscopy (AFM) [16,17,18]. However, the method of dynamic light scattering is also used to describe gel formation by pectin. It was used to study the gelation process of low-methoxy pectin influenced by an increase in pectin concentration and modification of temperature [19] or the addition of calcium ions [20]. The time correlation function data were analyzed to characterize this process. In this study, the mean hydrodynamic diameter of sodium carbonate-soluble pectin (named DASP) fraction particles and the aggregation index, determined on the basis of measurements of light scattering at one and two angles, respectively, were applied to evaluate the cross-linking process of this fraction influenced by its concentration. The aggregation index is used to describe protein aggregation [21]—up to that point in time, it had not been applied to characterize pectin cross-linking and gelation. Therefore, other studies, such as atomic force microscopy, viscosity and pH measurements, were executed to evaluate the cross-linking of the DASP fraction. AFM studies were performed to show changes in the nanostructure of pectin extracted using sodium carbonate with increasing concentration. Viscosity measurements are usually used to describe pectin cross-linking and gel formation [13], therefore, they were used to verify the results obtained from the analysis of the mean hydrodynamic diameter of particles and the aggregation index. pH measurements were also used to calculate the degree of hydrogen ion binding by the DASP fraction, which was dependent on the DASP fraction concentration. To the best of our knowledge, the relationship between the cross-linking of the DASP fraction and its concentration in pure water and at constant temperature has not been demonstrated yet. Our previous studies were connected with the cross-linking process of pectin extracted using sodium carbonate in different pH conditions [22] and in the presence of zinc ions [23], however, the investigation of the influence of other factors, such as the pectin concentration, on the above-mentioned process will enrich our knowledge about the properties of this pectin fraction.

The aim of this research was to characterize the influence of the DASP fraction concentration on its cross-linking process without the addition of salts or the modification of pH or temperature. An increase in the DASP fraction concentration in pure water at constant temperature resulted in pectin network formation, which was determined by the analysis of the dual-angle light scattering combined with the atomic force microscopy imaging, viscosity and pH measurements. 

## 2. Results and Discussion

### 2.1. Monosaccharide Composition and Methylesterification

The amount of uronic acids in sodium carbonate-soluble pectin fraction, isolated from “Golden Delicious” apples, was ~59 mol % of the total uronic acids content in alcohol-insoluble residue [22]. Therefore, this fraction had the highest amount of uronic acids, mainly galacturonic acid, in comparison to water- (WSP) and chelator-soluble pectin (CSP) fractions extracted, which was also noted for the DASP fraction from “Idared” apples [23]. The analysis of the monosaccharide composition of pectin extracted using sodium carbonate from apples cv. “Golden Delicious” indicated that homogalacturonan was the main pectin domain in the DASP fraction due to the presence of significant amounts of uronic acids, especially galacturonic acid (60.43 ± 0.20 mol %) [22]. Moreover, a large content of l-arabinose and d-galactose (33.29 ± 0.67 mol %) and the presence of l-rhamnose (3.10 ± 0.08 mol %) evidenced the occurrence of rhamnogalacturonan I, while a small amount of l-fucose (0.32 ± 0.03 mol %) may be interpreted as the presence of rhamnogalacturonan II in the DASP fraction.

A previously conducted analysis of FTIR spectra showed a very low absorbance value for a band at 1731 cm^−1^, characteristic of methyl esters, which indicated a very low degree of methylesterification of this fraction [22].

### 2.2. Nanostructure

**Figure 1:**
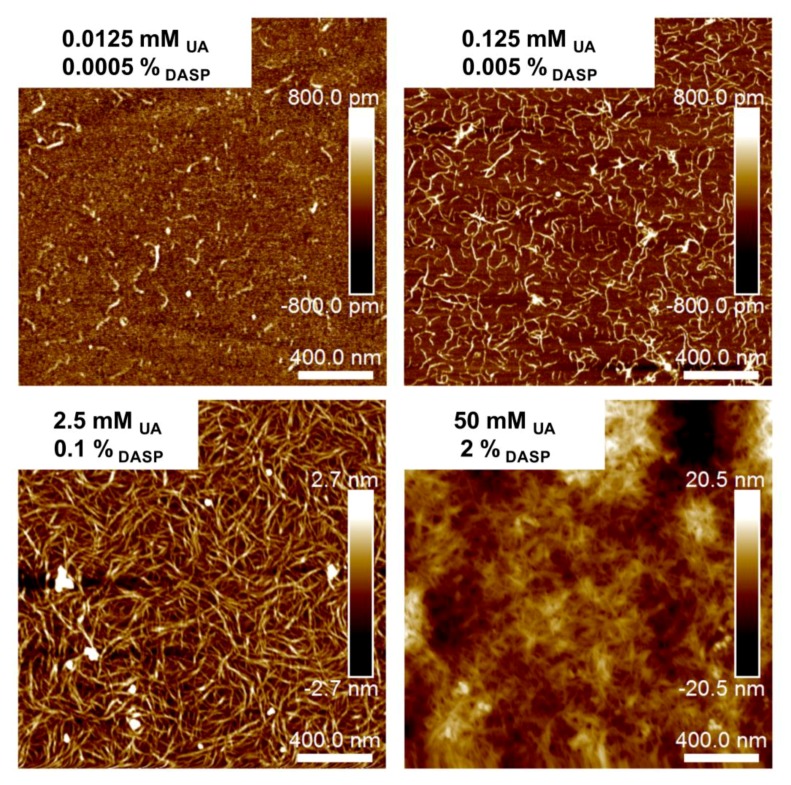
The atomic force microscopy (AFM) height images of 0.0005%, 0.005%, 0.1% and 2% sodium carbonate-soluble pectin (DASP) fraction samples and corresponding uronic acids (UA) concentration.

The values of the surface roughness parameters, determined on the basis of AFM image analysis, are presented in Table 1. Generally, an increase in the values of average roughness (S_a_) and root mean square roughness (S_q_) were observed with increasing DASP fraction concentration. At low DASP fraction concentration (C_DASP_ = 0.0005%), the single and separated unbranched chains are visible on the AFM image (Figure 1). At higher DASP fraction concentration (C_DASP_ = 0.005%), the extended and branched pectin chains were formed, however, the values of the surface roughness parameters were similar to those for the sample containing single chains (C_DASP_ = 0.0005%). The true branch points are characterized by the same height as that of the whole macromolecule [24,25]. These branch points were found at C_DASP_ = 0.005%. The branched chains were also present in the DASP fraction extracted from mung bean sprouts, strawberry and unripe tomato [24,26,27,28]. At the same concentration and lower pH (4), the DASP fraction formed a network on mica [22]. In the present study, the sample pH was 5.21 ± 0.01, which resulted in a higher dissociation degree of galacturonic acid units of the DASP fraction and stronger electrostatic repulsion between dispersed particles. The network formation and overlapping of chains on mica was observed at C_DASP_ = 0.1%, this was confirmed by a 5-fold increase in an average roughness and a root mean square roughness in relation to the lowest DASP fraction concentration (Table 1). With increasing pectin concentration, an increase in the surface roughness parameters connected with the formation and extension of the macromolecule network was observed. An effect of the sodium alginate concentration on the cross-linking process of this polysaccharide was investigated by Wang, Wan, Wang, Li, and Zhu [29]. At a sodium alginate concentration equal to 0.0005% (*w*/*v*), the short fibers separating from each other were noted, which is in agreement with the results presented for pectin here. An increase in alginate concentration of up to 0.002% (*w*/*v*) caused network formation, which was accompanied by an increase in the root mean square roughness [29]. A similar effect of the concentrations of low-methoxy pectin from apples on its nanostructure on mica was observed by Zareie, Gokmen, and Javadipour [30]. A gel-like structure was observed at concentration C_DASP_ = 2%, corresponding to about a 30-fold increase in surface roughness parameters. A similar structure was found on the AFM image of the hydrated gellan gel [18].

### 2.3. Particle Size and Aggregation Index (Ability of Light Transmittance)

**Figure 2:**
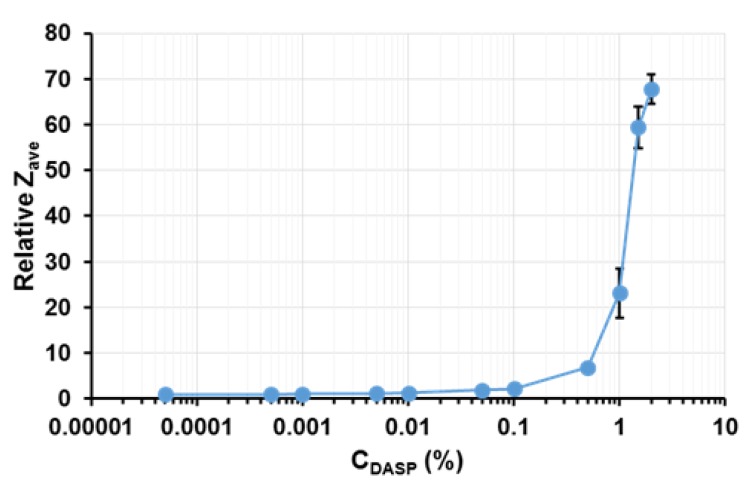
Relationship between the relative mean hydrodynamic diameter (relative Z_ave_) and the DASP fraction concentration (C_DASP_); the bars indicate standard deviation.

An increase in the relative mean hydrodynamic diameter (relative Z_ave_) was noted above the DASP fraction concentration C_DASP_ = 0.1% (Figure 2). This change in the size of dispersed particles corresponded to the pectin network formation observed using AFM (Section 2.2). An increase in polysaccharide concentration in solution affects the diffusion of its poly-ions [31] and Z_ave_ value obtained from dynamic light scattering (DLS) method [32]. At a polyelectrolyte concentration higher than about 0.001%, two diffusion behaviors may be noted, due to the occurrence of inter- and intramolecular interactions [33]. The fast diffusive mode is connected with the interaction between poly-ions and counter-ions forming counter-ion clouds around them [31]. The slow mode is related to the occurrence of cross-links between dipoles, formed as a result of the adsorption of counter-ions on polymer chains, which leads to aggregation. Generally, the values of the fast diffusion coefficient are constant at a polyelectrolyte concentration above 0.01%, while an increase in polyelectrolyte concentration causes a decrease in the slow diffusion coefficient [33]. Therefore, it may be concluded that in the range of the DASP fraction concentration above 0.1%, the larger influence on the value of the mean hydrodynamic diameter was slow diffusion, which indicates an occurrence of aggregates in samples and may confirm the formation of a pectin network. An increase in the mean hydrodynamic diameter with the polysaccharide concentration increasing from 0.2 to 0.6% (*w*/*v*) was also determined by other authors during sodium alginate and chitosan studies [34].

**Figure 3:**
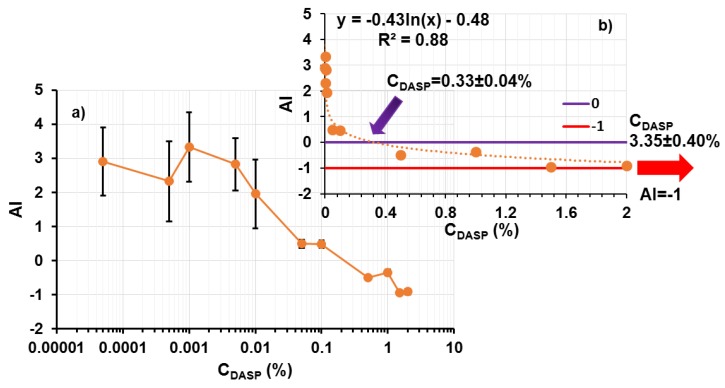
(**a**) The dependence of the aggregation index (AI) on the DASP fraction concentration (C_DASP_) and (**b**) the determination of the pectin concentration corresponding to AI = 0 and AI = −1; the bars indicate standard deviation.

The values of the aggregation index (AI) up to C_DASP_ = 0.01% were quite stable (Figure 3a). Above this concentration, a decrease in AI and a change in the AI sign from positive to negative were observed. This indicated the increasing difference in the forward and backward light scattering by the sample (Section 3.4) as a consequence of a change in the size and structure of particles dispersed in water as well as decreasing light transmittance. The obtained value of 0 AI reflects the same light scattering by the sample in all directions. In the case of the polysaccharide solution, it may be an effect of the formation of a homogeneous three-dimensional structure in bulk due to the cross-linking process. It was detected at a DASP fraction concentration of 0.33 ± 0.04% (Figure 3b). A further decrease in AI with the increasing content of pectin indicated that the light transmittance through the samples decreased, and so their turbidity increased. Huynh, Lerbret, Neiers, Chambin, and Assifaoui noted an increase in viscosity and turbidity, which was connected with the formation of a polygalacturonate network in the presence of divalent cations [35]. The value of AI = −1 means the lack of light transmittance through the sample and points to a well cross-linked sample. On the basis of the function equation fitted to the experimental data, the value of the DASP fraction concentration for AI = −1 was predicted to be 3.35 ± 0.40%.

The slight differences in results obtained from atomic force microscopy and dynamic light scattering are connected with different methods used to evaluate the cross-linking process of the DASP fraction. Moreover, in the case of the first method, the solid samples were prepared, while the liquid samples were studied using the second one. The changes to the aggregation index were found above C_DASP_ = 0.01%, which is close to the results obtained from the AFM image analysis. The ~2-fold increase in S_a_ and ~3-fold increase in S_q_ (in relation to the lowest concentration) was observed at C_DASP_ = 0.01 and C_DASP_ = 0.05.

### 2.4. Viscosity and Degree of Hydrogen Ion Binding

**Figure 4:**
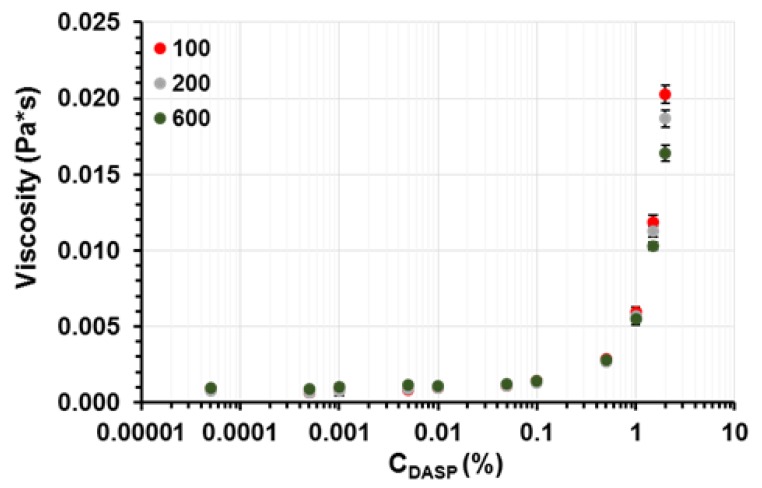
The dependence of viscosity on the DASP fraction concentration. Viscosity values were determined at different shear rates: γ = 100 (red circles), 200 (grey circles) and 600 s^−1^ (green circles). Mean values with standard deviations are given.

The changes in viscosity with increasing DASP fraction concentration were noted at different shear rates (Figure 4). An increase in viscosity was found above the DASP fraction concentration equal to 0.1%, which confirmed the results obtained from the dynamic light scattering method. This was a consequence of network formation [35]. An increase in pectin concentration caused a reduction in the distance between macromolecules, facilitating hydrogen bond formation. A similar effect of pectin concentration on viscosity was noted by Kar & Arslan and Ström et al. [13,36]. A rapid increase in the viscosity of low-methoxy pectin solutions was observed above its concentration equal to 0.1% *w*/*w* [13], which is close to the results obtained here. However, Hua, Wang, Yang, Kang, and Zhang noted a considerable increase in viscosity above the concentration of low-methoxy sunflower pectin equal to 1% (*w*/*w*) [37]. The differences between these data and the results presented here may be connected with the different chemical structure of the above-mentioned pectin and the DASP fraction studied.

**Figure 5:**
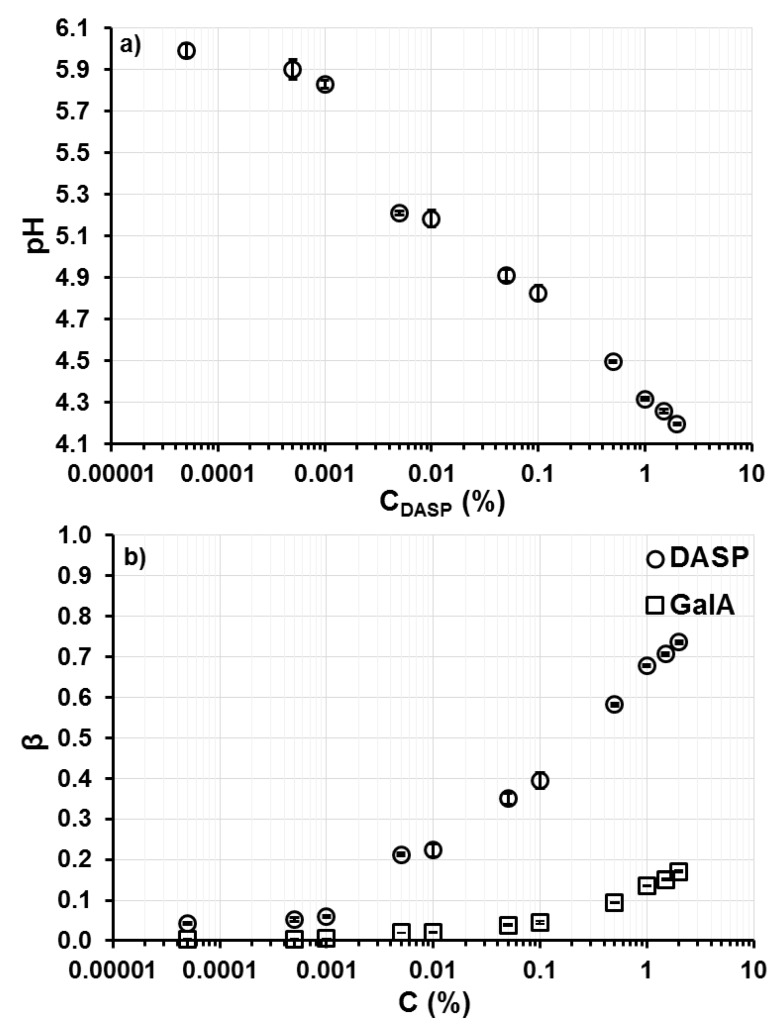
(**a**) Relationship between the pH of the samples and the DASP fraction concentration (C_DASP_) and (**b**) the dependence of the degree of hydrogen ion binding (β) on concentration (C) of the DASP fraction (circles) and galacturonic acid (squares); bars indicate standard deviation.

The pH measurements and determination of the hydrogen ion binding degree confirmed the participation of hydrogen bonds in the cross-linking process of the DASP fraction. An increase in pectin concentration from 0.00005% to 2% resulted in a pH decrease from 5.99 ± 0.03 to 4.20 ± 0.01 (Figure 5a). This was a consequence of the acidic character of the DASP fraction, which contains galacturonic acid (GalA) units possessing -COOH groups. The investigated fraction, extracted from apples using sodium carbonate, has weaker acidic character than GalA [22]. This leads to stronger binding of hydrogen ions by the DASP fraction than by GalA (Figure 5b). The degree of hydrogen ion binding by the DASP fraction was calculated on the basis of the apparent dissociation constant (pK_app_) of this fraction, determined using the measurements of electrophoretic mobility [22]. The pectin fraction was dissolved in water without the addition of salts, therefore pK_app_ is a more appropriate way to describe the dissociation process than the intrinsic dissociation constant, which is related to the dissociation of monomer units in a solution characterized by high ionic strength [38,39]. The pK_app_ of pectin with the degree of esterification DE = 0% was 4.1 [40], which is close to the results obtained for the DASP fraction. The analysis of the chemical composition of the DASP fraction revealed that its main component is galacturonic acid (60.43 ± 0.20 mol %) [22]. Therefore, a comparison of the hydrogen ion binding degree by GalA and the DASP fraction was determined. The difference in hydrogen ion binding degree increased with increasing GalA and DASP fraction concentration. At C_DASP_ = 0.5%, less than 50% of pectin carboxyl groups dissociated (β = 0.6) and the dissociation degree decreased to about 25% (β = 0.75) in a 2% pectin solution. For the same concentration range, the dissociation degree of galacturonic acid, which was calculated based on the pH of the DASP fraction solutions, was very high (about 90%) and it decreased to only 80%. With increasing concentrations of weak polyelectrolyte, the dissociation degree decreases [41]. In the studied case, this decrease facilitated the formation of hydrogen bonds between pectin macromolecules. Three types of hydrogen bonds, formed by the carboxyl groups of pectic acid, are suggested on the basis of X-ray diffraction analysis [42]. The intramolecular bond may be formed between a carboxyl group in one GalA unit and a hydroxyl group at C(2) of an adjacent unit in the same chain. The intermolecular bond may occur between a carboxyl group of one chain and a hydroxyl group at C(3) of a neighboring chain. The two carboxyl groups from two neighboring chains may also be involved in the formation of a hydrogen bond. However, at least one of these groups may not be ionized to be the hydrogen-bond donor. Moreover, hydrogen bonds may be formed between the hydroxyl groups of LM pectin [43,44].

Considering that the cross-linking of the DASP fraction was studied using pure water as a dispersant (without salt addition), hydrogen bond formation, facilitated by the pH decrease and an increase in the hydrogen ion binding degree, was the most important factor influencing pectin network formation.

## 3. Materials and Methods

### 3.1. Isolation of Cell Wall Material and Pectin Fractionation

Apples cv. ”Golden Delicious” were harvested at the optimum maturity time and purchased from the local orchard. Cell wall material was extracted according to the protocol of Renard [45], with slight modifications, as an alcohol-insoluble residue (AIR). Ethanol (~70%) was added to the apple pulp. This was mixed for 0.5–1 h, filtered on a paper filter, and ethanol was again added to the residue. This was repeated until the removal of sugars, which was confirmed by the test of Dubois, Gilles, Hamilton, Rebers, and Smith [46]. Finally, the residue was mixed with 96% ethanol, and, after filtration, this action was repeated with acetone. The pulp was put into the oven set to 40 °C.

Pectin fractionation was executed using the following solvents: H_2_O (deionized), CDTA—trans-1,2-diaminocyclohexane-*N*,*N*,*N′*,*N′*-tetraacetic acid (0.05 M, pH 6.5) and Na_2_CO_3_ (0.05M) with NaBH_4_ (0.02M) [23,47]. As a result of this sequential extraction, water-soluble pectin (WSP), chelator-soluble pectin (CSP) and sodium carbonate-soluble pectin or diluted alkali-soluble pectin (DASP) were obtained, respectively. 

The dialysis of the DASP fraction (ZelluTrans/Roth^®^ dialysis membranes, MWCO 3500; Carl Roth GmbH & Co. KG, Karlsruhe, Germany) and then lyophilization were performed. The freeze-dried DASP fraction was then stored in a vacuum dryer to prevent water absorption. 

The determination of monosaccharide composition and degree of methylesterification was performed and analyzed previously [22]. 

### 3.2. Preparation of Samples

The samples containing from 0.00005% to 2% (*w*/*v*) fraction of pectins, which are soluble in diluted alkali solution, were prepared by dissolving the appropriate weight of lyophilized pectin in deionized water (water conductivity 2 ± 0.06 mS/m). The uronic acid concentration corresponding to the above pectin content ranged from 0.00125 to 50 mM. The samples were prepared a day before measurements and they were conditioned with gentle mixing for 24 h at 20 °C.

### 3.3. AFM Imaging and Quantitative Analysis

The DASP fraction samples were deposited on freshly cleaved mica, spread using a spin-coater SPIN150i (SPS-EUROPE, Putten, The Netherlands) and then dried in a desiccator overnight at 22 °C and 15% relative humidity. A Multimode 8 equipped with a Nanoscope V controller (Bruker, Billerica, MA, USA) were used to imaging in the semiautomatic high-speed tapping mode. A silicon nitride cantilever ScanAsyst-Air (Bruker, Billerica, MA, USA) was used for scanning. All the AFM studies were performed in ambient air at a RH = 25–30% and room temperature. For each sample, five height images (scan size 2 × 2 μm) were collected with a scan rate of 2.0 Hz and a resolution of 512 × 512 points. The quantitative analysis of AFM height images, including surface roughness parameters, such as the average roughness (S_a_) and the root mean square roughness (S_q_) [48], was performed using SPIP 6.2.0 software (Image Metrology, Hørsholm, Denmark).

### 3.4. Particle Size and Aggregation Index Determination in Pectin Solutions

A Zetasizer Nano ZS (Malvern Instruments Ltd., Malvern, UK) with the source of red laser light was applied to determine the particle size (Z_ave backward_) using the method of dynamic light scattering (DLS), according to the international standard [32]. Z_ave backward_ was determined based on the measurements at an angle of 173°, while the aggregation index (AI) was calculated using also the results obtained from forward scattering at an angle of 12.8° (dual-angle scattering), according to this equation [21],

AI = (Z_ave forward_/Z_ave backward_) − 1 (1)

where Z_ave forward_/Z_ave backward_—ratio of particle sizes obtained from forward/backward light scattering of the particles dispersed in liquid.

Four measurements were executed for each sample at 20 °C.

The mean hydrodynamic diameter means the sphere diameter, including a hydration layer, and performing Brownian motion similar to the studied particle [32]. The relative value of this parameter for each sample was calculated in relation to the value of the mean hydrodynamic diameter obtained for the lowest DASP fraction concentration.

### 3.5. Viscosity Measurements

Viscosity measurements were conducted using a Discovery Hybrid Rheometer (HR-1) with a parallel plate geometry (d = 20 mm) delivered by TA Instruments (New Castle, PA, USA) at a shear rate γ = 100, 200, and 600 s^−1^ at a temperature of 20 °C.

### 3.6. pH Determination and Calculations

The pH (±0.01 pH) of the DASP fraction solutions was measured using the Oakton pH Spear tester (Osprey Scientific Inc., Edmonton, AB, Canada) in 3 repetitions at 20 (±1) °C. On the basis of the obtained pH values and the known dissociation constants (pK_app,DASP_ = 4.64 from [22], and pK_a,GalA_ = 3.51 from [49]), the hydrogen ion binding degree (β) was calculated [49]:

pH = pK_a_ + log(α/1 − α) (2)

β = 1 − α(3)

## 4. Conclusions

The determination of the beginning of pectin network formation is essential in the investigation of the pectin cross-linking and gel formation, which are used in the food and biomedical industry and may be useful in agriculture. 

The aggregation index has been applied for the first time to describe the polysaccharide cross-linking process. The change in the aggregation index sign from positive to negative indicated the formation of a pectin network, which was confirmed by the results obtained from viscosity measurements and an atomic force microscope. Thus, the aggregation index may be a good indicator of macromolecule network formation. 

An increase in sodium carbonate-soluble pectin concentration results in the lowering of pH, and, therefore, in an increase in the hydrogen ion binding and the shortening of intermolecular distance. These phenomena affect the cross-linking of sodium carbonate-soluble pectins in pure water via hydrogen bond formation.

## Figures and Tables

**Table 1:** The surface roughness parameters of AFM images—average roughness (S_a_) and root mean square roughness (S_q_). The minimum and maximum values of these parameters are given.

C_DASP_ (%)	S_a min_	S_a max_	S_q min_	S_q max_
0.00005	0.1029	0.1092	0.1535	0.1691
0.0005	0.0919	0.1106	0.1441	0.1500
0.001	0.1487	0.2100	0.2145	0.3630
0.005	0.1182	0.1291	0.1754	0.2155
0.01	0.2021	0.2451	0.3778	0.5186
0.05	0.2107	0.2317	0.4085	0.4957
0.1	0.5425	0.5680	0.7975	0.8733
0.5	1.3817	1.4408	1.8101	2.0064
1	2.0590	2.7888	2.7928	3.9249
1.5	2.4921	2.8095	3.3201	3.7959
2	3.1350	3.6414	4.1914	4.9073
